# Prognostic Relevance of Gradient and Flow Status in Severe Aortic Stenosis

**DOI:** 10.3390/jcm13206113

**Published:** 2024-10-14

**Authors:** Eduardo Pozo Osinalde, Juan Ramón Bravo Domínguez, Lina De Lara Fuentes, Pedro Marcos-Alberca, José Juan Gómez de Diego, Carmen Olmos Blanco, Patricia Mahia Casado, María Luaces Mendez, Luis Collado Yurrita, Manuel Carnero-Alcázar, Pilar Jiménez-Quevedo, Luis Nombela-Franco, Julián Pérez-Villacastín

**Affiliations:** 1 Cardiology Department, Cardiovascular Institute, Hospital Clínico San Carlos, Instituto de Investigación Sanitaria del Hospital Clínico San Carlos (IdISSC), 28040 Madrid, Spain; juanrbra@ucm.es (J.R.B.D.); linadelara89@gmail.com (L.D.L.F.); pedro.marcosalberca@salud.madrid.org (P.M.-A.); josejuan.gomez@salud.madrid.org (J.J.G.d.D.); carmen.olmos@salud.madrid.org (C.O.B.); patricia.mahia@salud.madrid.org (P.M.C.); mluaces@salud.madrid.org (M.L.M.); pjimenezq@salud.madrid.org (P.J.-Q.); luis.nombela@salud.madrid.org (L.N.-F.); julian.perez-villacastin@salud.madrid.org (J.P.-V.); 2 Medicine Department, Complutense University, 28040 Madrid, Spain; lcollado@ucm.es; 3 Cardiac Surgery Department, Cardiovascular Institute, Hospital Clínico San Carlos, Instituto de Investigación Sanitaria del Hospital Clínico San Carlos (IdISSC), 28040 Madrid, Spain; manuel.carnero@salud.madrid.org

**Keywords:** severe aortic stenosis, echocardiography, paradoxical low-flow low-gradient, prognosis

## Abstract

**Background**: Severe aortic stenosis (AS) may present with different flow, gradient and left ventricular ejection fraction (LVEF) patterns. Paradoxical low-flow low-gradient (PLF-LG) severe AS has a specific clinical profile, but its prognosis and management remain controversial. Our aim is to evaluate the impact of different AS patterns in the incidence of major clinical events. **Methods**: A retrospective observational study was carried out on all the consecutive patients diagnosed with severe AS at our tertiary hospital centre in 2021. Echocardiographic measurements were carefully reviewed, and patients were classified following current guidelines into four categories: high gradient (HG), concordant low-flow low-gradient (CLF-LG), paradoxical low-flow low-gradient (PLF-LG) and normal-flow low-gradient (NF-LG). The baseline characteristics and clinical events (heart failure admission, intervention and death) at 1-year follow-up were collected from medical records. The association between categories and events was established using Student’s *t* test or ANOVA as required. **Results**: 205 patients with severe AS were included in the study (81 ± 10 years old, 52.7% female). Category distribution was as follows: HG (138, 67.3%), PLF-LG (34, 19.8%), CLF-LG (21, 10.2%) and NF-LG (12, 5.9%). During the follow-up, 24.8% were admitted due to heart failure, 68.3% received valve replacement (51.7% TAVR) and 22% died. Severe tricuspid regurgitation was more frequent in patients with PLF-LG than in HG AS (14.7% vs. 2.2%; *p* < 0.01). Despite no differences in intervention rate, more patients with PLF-LG (32.4% vs. 15.9%; *p* = 0.049) died during the evolution. **Conclusions**: The PLF-LG pattern was the second most common pattern of severe AS in our cohort, and it was related to a higher mortality with no differences in intervention rate. Thus, this controversial category, rather than being underestimated, should be followed closely and considered for early intervention.

## 1. Introduction

Aortic stenosis (AS) is the first indication of valvular intervention in patients in developed countries [1]. The ageing of the population has led to a progressive increase in this pathology prevalence and may hinder the diagnosis of its clinical relevance. Transthoracic echocardiography (TTE) remains the main diagnostic tool used for the detection and grading of AS based on transvalvular peak velocity (Vmax) and mean gradient (ΔPm) as well as aortic valve area (AVA) determination. Severe AS has been defined as an AVA < 1 cm^2^ and classified according to the interaction of Vmax, ΔPm, flow status and left ventricular ejection fraction (LVEF).

Paradoxical low-flow low-gradient (PLF-LG) severe AS is considered in the presence of Vmax < 4 m/s and ΔPm < 40 mm Hg and a low flow stroke volume index (SVi) < 35 mL/m^2^, despite preserved systolic function (LVEF ≥ 50%). Apart from the diagnostic challenges of this entity [2], significant differences have been noted in its epidemiology, clinical profile and evolution. Patients with so-called “classic AS” are younger and more frequently male, whereas in PLF-LG AS, the prevalence of elderly patients and comorbidities, such as coronary artery disease, hypertension and atrial fibrillation, is higher [3]. Regarding echocardiographic features, the latter group shows smaller LV volumes with greater wall thicknesses, resulting in slightly lower LVEF and decreased SVi [4,5,6]. On top of these differences and an increased valvulo-arterial impedance [4], depressed myocardial deformation, determined as a reduced global longitudinal strain, not only may be the basis of this entity but has also demonstrated prognostic impact [7]. Moreover, a wide QRS [8] and significant tricuspid regurgitation [9] have also been described as independent predictors of mortality. Finally, there are significant discordances regarding the prognosis of PLF-LG AS. Since the first description, Pibarot and colleagues have argued that this entity carries out a poor prognosis [4], which has been corroborated in multicenter studies [10]. Other authors [11,12] advocate that the related mortality is lower than in “classic AS”, with some even precluding that it is closer to moderate AS [3]. Regarding valvular intervention, although surgical aortic valve replacement (SAVR) is less frequently performed in PLF-LG AS, it is associated with improved prognosis [13]. Moreover, transcatheter aortic valve replacement (TAVR) seems to be superior to AVR in this subgroup of patients [14]. In the same way, the current guideline [15,16] recommendation of intervention for PLF-LG AS is weaker than for the classical form.

In light of the above, our aim is to evaluate the prognostic relevance of gradient and flow status patterns in an unselected current population of patients with severe aortic stenosis and find predictors in PLF-LG.

## 2. Methods

### 2.1. Study Population

All the studied patients with a definitive diagnosis of severe AS according to ongoing recommendations [2] were retrospectively selected from the echocardiographic database at our tertiary hospital in 2021. In those cases with multiple studies, the first one was selected. Baseline characteristics and clinical events (heart failure admission, intervention and death) at 1-year follow-up were collected from digital medical records.

### 2.2. Echocardiography Studies Acquisition and Analysis

**Figure 1:**
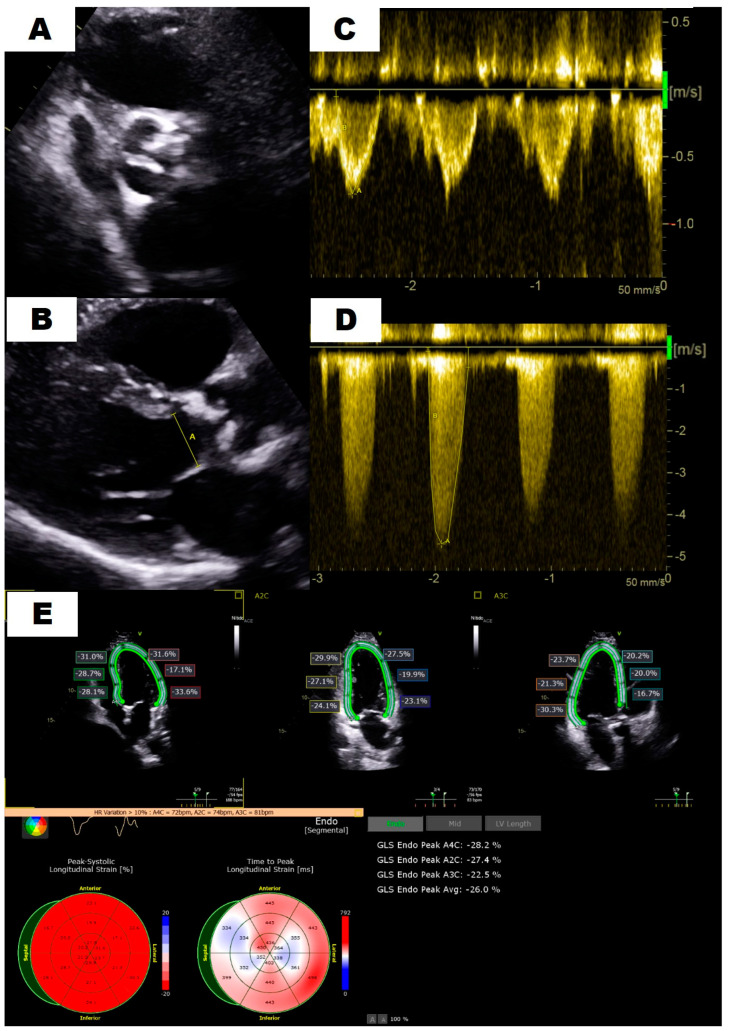
Comprehensive evaluation of aortic stenosis severity. An initial morphologic evaluation of the aortic valve in a short axis view (**A**) is crucial for careful measurement of left ventricular outflow tract diameter (**B**). Pulsed (**C**) and continuous (**D**) Doppler allow for the estimation of flow and aortic valve gradient, respectively. Finally, a comprehensive evaluation may include left ventricular strain analysis (**E**).

**Figure 2:**
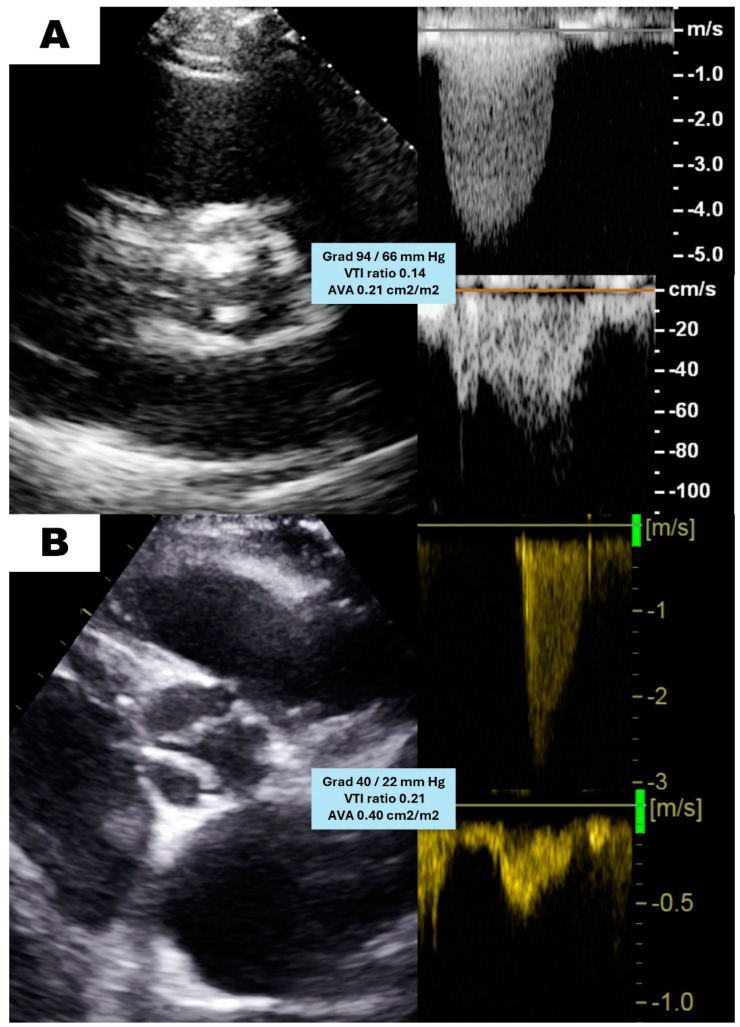
Echocardiographic features of the different patterns of aortic stenosis. Morphological and functional differences between high gradient (**A**) and paradoxical low-flow low-gradient (**B**) aortic stenosis. Please note that both forms result in a severely reduced aortic valve area regardless of the gradients.

The majority of the exams were performed in Philips iE33 or EPIC cardiac ultrasound equipment with a 2–4 MHz transducer (Philips, Andover, MA, USA) following current recommendations [17]. The studies were retrieved from PACS and carefully reviewed blindly by 3 cardiac imaging experts. Once the diagnosis of severe AS was confirmed by consensus (Figure 1), the cases were classified according to gradients, flow status and LVEF as stated in the guidelines [15,16] into four categories: high gradient (HG), concordant low-flow low-gradient (CLF-LG), paradoxical low-flow low-gradient (PLF-LG) and normal-flow low-gradient (NF-LG). Other relevant echocardiographic parameters, such as LV dimensions and ejection fraction, TAPSE or the grading of other coexisting valvulopathies, were collected as well (Figure 2).

### 2.3. Statistical Analysis

Qualitative variables are presented as absolute numbers and percentages and quantitative variables as mean ± standard deviation or median [interquartile range] as appropriate. Qualitative data are compared using the chi-square or Fisher’s exact test. The association between categories and events was established using Student’s *t* test or ANOVA as required. Differences were considered statistically significant when the bilateral *p* value was <0.05. SPSS version 23.0 (SPSS Inc., Chicago, IL, USA) was used for the statistical analysis.

## 3. Results

**Figure 3:**
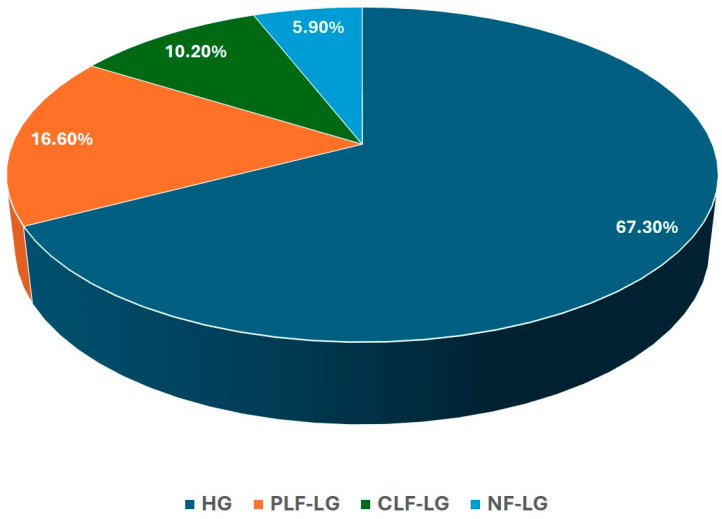
Prevalence of severe aortic stenosis flow gradient patterns. HG: high gradient; PLF-LG: paradoxical low-flow low-gradient; CLF-LG: concordant low-flow low-gradient; NF-LG: normal-flow low-gradient.

In 2021, 205 patients were diagnosed with severe AS in our tertiary centre (81 ± 10 years old, 52.7% female). They were classified with respect to AS category (Figure 3): HG (138, 67.3%), PLF-LG (34, 16.6%), CLF-LG (21, 10.2%) and NF-LG (12, 5.9%). For the purpose of the present study, we solely compared patients with HG and PLF-LG AS. Baseline clinical characteristics are described in Table 1. Despite similar age, gender distribution or cardiovascular risk factor prevalence, PLF-LG AS showed higher surgical risk (EuroScore 2.7 vs. 4; *p* = 0.044). Moreover, there was a difference in tricuspid regurgitation (TR), with a higher prevalence of severe insufficiency (2.2 vs. 14.7%; *p* = 0.001).

Echocardiographic characteristics are collected in Table 2. As expected, transvalvular gradients were greater in HG AS, whereas both the velocity time integral (VTI) ratio (0.22 vs. 0.24; *p* = 0.04) and aortic valve area (AVA) (0.66 vs. 0.71 cm^2^; *p* = 0.048) were slightly higher in PLF-LG AS. Although there were no differences in left ventricular ejection fraction (LVEF), the stroke volume indexed (SVi) was significantly lower in PLF-LG AS (39.6 vs. 28.5 mL/m^2^; *p* <0.001). Notably, these patients showed less thickened left ventricular walls with similar end-diastolic diameter.

Regarding cardiovascular events during a median follow-up of 23 (IQR: 19–25) months (Table 3), the intervention rate and time-to-intervention rate were similar in the groups. The most frequent procedure was TAVR (49.4%), and neither demonstrated differences in intervention type distribution. The incidence of admission due to heart failure or any cause was similar in both groups. Death during the follow-up was significantly higher in patients with PLF-LG AS (15.9 vs. 32.4%; *p* = 0.029), justified by a trend to greater mortality among those treated medically (32.6 vs. 57.1%; *p* = 0.12).

## 4. Discussion

In our study, PLF-LG was the second most common pattern in an unselected series of patients diagnosed with severe AS, accounting for one in five cases with similar main demographic characteristics. The lower SVi in this group was not explained by differences in LVEF or left ventricular structure. Finally, these patients showed higher mortality despite a similar rate of intervention.

Since its first description, PLF-LG prevalence and its clinical profile have been a source of controversy. Firstly, it was described by Hachicha et al. [4] as a frequent presentation (35%) of AS. However, subsequent studies have shown a variable proportion of PLF-LG ranging from 3% [18] to 35% [7], including a catheterization-based study accounting for 26% [6]. Our prevalence is close to the later series, reinforcing the methodic echocardiographic evaluation of our cases. Curiously, we did not find sex differences in the distribution of PLF-LG AS while this entity has been associated with the female gender. Moreover, the distribution of cardiovascular risk factors and ischemic heart disease was similar between groups. These differences may be explained by discordances in reference populations, which is probably justified by the more advanced age of our cohort.

Regarding echocardiographic features of PLF-LG, this entity has been classically associated with small LV cavities with markedly thickened walls [4]. The subsequent diastolic dysfunction has been argued as a possible mechanism of discordance between preserved LVEF and reduced SVi [19]. However, in our series, there were no differences in LV dimensions, and, rather, there was thicker myocardium among HG AS patients. Among the other imaging characteristics previously described [18], TR severity was solely related to PLF-LG in the present population. This valvulopathy has been previously underestimated, but it is common in the general population and closely related to ageing [20]. Moreover, it has been related to higher mortality in CLF-LG AS [9]. Dahou et al. demonstrated that TR ≥ 2 was an independent predictor of all-cause and cardiovascular death in this group of patients, as previously reported for chronic heart failure [21]. This may be explained by the association of TR with reduced stroke volume, which has shown a prognostic impact in AS [22]. TR is also related to right ventricular dilation and dysfunction, another main cardiovascular predictor [23], and might even mask the presence of reduced right ventricular systolic function [24].

PLF-LG AS has been associated with a higher surgical risk [25] derived from coexisting comorbidities and marked concentric LV hypertrophy [26]. Consequently, this group showed a significantly greater EuroScore in our series. Despite that, there were no differences in the intervention rate, type of replacement, or waiting time between groups. TAVR was the leading intervention in both patterns. In any case, PLF-LG patients suffered higher mortality during the follow-up, mainly related to conservative treatment. These results are concordant with previous studies [6,22,27] and reinforce that this entity has a worse prognosis and should undergo early intervention even in the presence of high surgical risk.

### Limitations

Some limitations need to be addressed in relation to the present work. Firstly, its retrospective design precludes the perfect control of all the variables related to patient selection and prognosis. However, regarding population, we collected consecutive cases from our echocardiography laboratory, and our PLF-LG prevalence is similar to the largest published cath-based diagnosis series [6]; therefore, we think it may mirror a real-life daily cohort of patients with severe AS. Moreover, baseline heart rhythm could not be confirmed in all the patients, so these data are missing in the analysis. Certainly, atrial fibrillation prevalence has been previously reported to be higher in PLF-LG AS patients but with no evidence of worse outcome [6]. Unfortunately, these findings could not be corroborated in the present study. On the other hand, some aspects previously related to the pathophysiology of PLF-LG, such as systemic hypertension [28], global longitudinal strain [29] or myocardial fibrosis [30], are missing. Unfortunately, blood pressure measurement at the time of the echocardiography was not available in the records. Myocardial deformation was not evaluated because studies were acquired with different vendor machines, and in some of them, image quality was not enough for speckle tracking analysis. For the last variable, cardiac magnetic resonance is not performed by the protocol for AS evaluation at our centre, so fibrosis could not be assessed. Nevertheless, we think that the aforementioned parameters have shown a more relevant role in PLF-LG diagnosis than in prognosis. In any case, we consider that the aim of our paper is to contrast previous findings, and our conclusions still need to be corroborated with larger prospective studies.

## 5. Conclusions

Our work highlights that PLF-LG is a common form of severe AS in an unselected population. Apart from potential differences in LV geometry, tricuspid regurgitation should be considered a potential coexisting valvulopathy. This entity has higher mortality in the follow-up but benefits from intervention equally to HG AS. Therefore, an early aortic valve replacement should be pursued in patients with PLF-LG AS regardless of their higher surgical risk.

## Figures and Tables

**Table 1:** Baseline clinical and echocardiographic characteristics.

	All (n = 172)	HG (n = 138)	PLF-LG (n = 34)	*p*
Age	81 ± 10	80.7 ± 10.2	82 ± 9.3	0.51
Female	95 (55.2%)	79 (57.2%)	16 (47.1%)	0.337
CV risk factors:				
-HTN	143 (83.1%)	113 (81.9%)	30 (88.2%)	0.453
-DM	73 (42.4%)	56 (40.6%)	17 (50%)	0.339
-Dyslipidemia	120 (69.8%)	98 (71%)	22 (64.7%)	0.533
-Smoker	50 (29.1%)	38 (27.5%)	12 (35.3%)	0.402
Ischemic heart disease	62 (36.0%)	46 (33.3%)	16 (47.1%)	0.164
MR:				0.773
-None	42 (24.4%)	34 (24.6%)	8 (23.5%)	
-Mild	94 (54.7%)	77 (55.8%)	17 (50%)	
-Moderate	30 (17.4%)	23 (16.7%)	7 (20.6%)	
-Severe	6 (3.5%)	4 (2.9%)	2 (5.9%)	
AR:				0.752
-None	84 (48.8%)	68 (49.3%)	16 (47.1%)	
-Mild	59 (34.3%)	46 (33.3%)	13 (38.2%)	
-Moderate	25 (14.5%)	20 (14.5%)	5 (14.7%)	
-Severe	4 (2.3%)	4 (2.9%)	0 (0%)	
TR:				0.001
-None	57 (33.1%)	49 (35.5%)	8 (23.5%)	
-Mild	82 (47.7%)	70 (50.7%)	12 (35.3%)	
-Moderate	25 (14.5%)	16 (11.6%)	9 (26.5%)	
-Severe	8 (4.7%)	3 (2.2%)	5 (14.7%)	
PHT:				0.081
-None	96 (55.8%)	83 (60.1%)	13 (38.2%)	
-Mild	36 (20.9%)	28 (20.3%)	8 (23.5%)	
-Moderate	23 (13.4%)	16 (11.6%)	7 (20.6%)	
-Severe	17 (9.9%)	11 (8%)	6 (17.6%)	
TAPSE < 17 mm	24 (14%)	17 (12.3%)	8 (23.5%)	0.097
GFR (mL/min)	65 [49.3–81]	66 [50–82.3]	63.5 [35–78.3]	0.123
EuroScore	2.8 [1.6–5.2]	2.7 [1.6–5.2]	4 [2.4–5.1]	0.044

CV: cardiovascular; HTN: hypertension; DM: diabetes mellitus; MR: mitral regurgitation; AR: aortic regurgitation; TR: tricuspid regurgitation; PHT: pulmonary hypertension; TAPSE: tricuspid annular plane systolic excursion.

**Table 2:** Echocardiographic features of aortic stenosis.

	All (n = 172)	HG (n = 138)	PLF-LG (n = 34)	*p*
Maximum velocity (m/s)	4.3 ± 0.6	4.5 ± 0.4	3.4 ± 0.4	<0.001
Mean gradient (mm Hg)	45 ± 13.3	49.3 ± 10.8	27.5 ± 6	<0.001
Aortic VTI (cm)	99.2 ± 20.2	105.1 ± 16.9	75.3 ± 14.1	<0.001
LVOT VTI (cm)	21.9 ± 8	22.9 ± 8.4	17.7 ± 4.2	<0.001
VTI ratio	0.22 ± 0.06	0.22 ± 0.06	0.24 ± 0.05	0.04
AVA (cm^2^)	0.67 ± 0.17	0.66 ± 0.18	0.71 ± 0.13	0.048
SV index (mL/m^2^)	37.4 ± 10.5	39.6 ± 10.3	28.5 ± 4.8	<0.001
LVEF (%)	62.3 ± 9.2	62 ± 9.7	63.6 ± 7.2	0.379
IVS thickness (cm)	1.31 ± 0.28	1.34 ± 0.27	1.22 ± 0.3	0.035
PW thickness (cm)	1.18 ± 0.23	1.2 ± 0.21	1.12 ± 0.27	0.069
LVEDD (cm)	4.35 ± 0.75	4.32 ± 0.77	4.47 ± 0.65	0.303

VTI: velocity time integral; LVOT: left ventricular outflow tract; AVA: aortic valve area; SV: stroke volume; LVEF: left ventricular ejection fraction; IVS: interventricular septum; PW: posterior wall; LVEDD: left ventricular end-diastolic diameter.

**Table 3:** Clinical events during the follow-up period.

	All (n = 172)	HG (n = 138)	PLF-LG (n = 34)	*p*
Any intervention	115 (66.9%)	95 (68.8%)	20 (58.8%)	0.266
Intervention type:				0.619
-TAVR	85 (49.4%)	71 (51.4%)	14 (41.2%)	
-Biological SAVR	27 (15.7%)	22 (15.9%)	5 (14.7%)	
-Mechanical SAVR	3 (1.7%)	2 (1.4%)	1 (2.9%)	
Time to intervention (months)	0.5 [0–4]	0 [0–4]	2 [0–3]	0.638
Any admission	78 (45.6%)	61 (44.5%)	17 (50%)	0.566
HF admission	39 (22.7%)	29 (21%)	10 (29.4%)	0.295
Death	33 (19.2%)	22 (15.9%)	11 (32.4%)	0.029
Time to event	2 [0–8]	3 [0–9]	0 [0–7]	0.345

TAVR: transcatheter aortic valve replacement; SAVR: surgical aortic valve replacement; HF: heart failure.

## Data Availability

Research data are not publicly available due to privacy but may be partially shared if required.
